# Supplementary material for: Spatial and temporal modeling of breast cancer mortality in Kansas: An R-INLA approach
Source: PLoS One. 2026 Apr 29;21(4):e0347607. doi: 10.1371/journal.pone.0347607 (PMC13127976; doi:10.1371/journal.pone.0347607)
Supplement: S1 File — (DOCX) [file pone.0347607.s001.docx]

**S1:** Descriptive statistics of all covariates within each county in Kansas over 2018-2021: Mean (Standard Deviation)

| **County** | **Cases** | **Population** | **Percent Female** | **Binge Drinking** | **Smoking** | **Diabetes** | **Obese** | **PCP** | **CARR** |
| --- | --- | --- | --- | --- | --- | --- | --- | --- | --- |
| Allen | 1.5 (0.58) | 12,478.25 (74.55) | 50.6 (0.18) | 15.63 (0.62) | 19.3 (0.64) | 9.78 (0.53) | 34.98 (2.85) | 128.75 (21.05) | 0.315341 |
| Anderson | 2 (0.82) | 7,839.5 (20.24) | 49.8 (0.67) | 15.85 (0.76) | 19.73 (0.26) | 7 (0.93) | 31.03 (1.27) | 66.75 (6.18) | 0.331937 |
| Atchison | 0.75 (0.5) | 16,201.5 (105.99) | 51.35 (0.13) | 17.2 (0.8) | 18.7 (1.53) | 7.65 (1.19) | 32.28 (3.77) | 108.5 (12.4) | 0.283181 |
| Barber | 0.75 (0.96) | 4,300 (173.96) | 48.93 (0.33) | 14.73 (0.63) | 19.38 (0.98) | 6.73 (0.28) | 27.83 (1.67) | 117.75 (8.92) | 0.515224 |
| Barton | 2.5 (2.08) | 25,632.25 (354.92) | 50.4 (0.18) | 15.53 (0.95) | 18.78 (0.9) | 9.73 (1.02) | 31.25 (2.57) | 141.5 (17.41) | 0.407406 |
| Bourbon | 1.75 (2.22) | 14,434 (120.16) | 50.43 (0.28) | 15.08 (0.5) | 19.53 (1.14) | 9.65 (1.17) | 38.3 (2.12) | 123.75 (15.46) | 0.320055 |
| Brown | 2.25 (1.5) | 9,527.25 (72.12) | 50.45 (0.26) | 15.35 (0.72) | 19.3 (0.8) | 9.08 (0.31) | 34.68 (3.94) | 76 (23.45) | 0.311337 |
| Butler | 7.25 (1.71) | 67,279.25 (530.24) | 49.3 (0.46) | 16.75 (0.99) | 16.93 (1.16) | 8.55 (0.61) | 36.48 (1.91) | 52.25 (6.45) | 0.290152 |
| Chase | 0.75 (0.96) | 2,607.75 (30.29) | 48.25 (0.73) | 15.35 (0.65) | 16.8 (0.84) | 7 (0.67) | 23.83 (1.45) | 38 (0.82) | 0.362594 |
| Chautauqua | 1.75 (1.71) | 3,329.5 (64.53) | 48.58 (0.36) | 14.23 (0.36) | 20.88 (0.79) | 6.95 (0.42) | 23.9 (4.26) | 274.25 (28.76) | 0.395526 |
| Cherokee | 3.25 (0.96) | 19,614.25 (434.92) | 50.45 (0.31) | 15.48 (0.6) | 20.55 (0.7) | 9.83 (0.72) | 32.9 (4.18) | 159 (6.48) | 0.293012 |
| Cheyenne | 0 (0) | 2,637.25 (19.09) | 49.3 (0.24) | 14.53 (0.38) | 16.93 (0.74) | 6.73 (0.43) | 22.78 (1.86) | 198.25 (21.85) | 0.577997 |
| Clark | 0.5 (1) | 1,980.5 (17.6) | 50.65 (0.17) | 15.75 (0.93) | 15.78 (0.62) | 6.4 (0.7) | 25.2 (1.26) | 314 (47.45) | 0.624515 |
| Clay | 0.75 (0.96) | 8,057.75 (64.81) | 49.93 (0.36) | 16 (0.74) | 16.55 (0.49) | 9.55 (0.39) | 32.25 (2.96) | 62.5 (10.21) | 0.320812 |
| Cloud | 0.75 (0.5) | 8,875.75 (137.05) | 49.78 (0.72) | 15.93 (0.78) | 18.1 (0.71) | 7.78 (0.4) | 31.4 (2.09) | 74.25 (15.65) | 0.375906 |
| Coffey | 0.5 (0.58) | 8,279 (87.93) | 50.38 (0.26) | 15.63 (0.51) | 17.38 (0.51) | 8.45 (1.56) | 30.73 (3.86) | 82.5 (12.01) | 0.318839 |
| Comanche | 0 (0) | 1,696.25 (29.74) | 51.2 (0.18) | 15.45 (0.38) | 15.68 (0.73) | 7.2 (0.27) | 23.13 (2.45) | 173.5 (4.43) | 0.565958 |
| Cowley | 9.25 (4.03) | 3,4805.75 (278.16) | 49.68 (0.05) | 16.35 (1.13) | 19.28 (0.86) | 9.28 (1.2) | 36.6 (1.34) | 71.5 (11.68) | 0.322672 |
| Crawford | 6 (2.58) | 38,928.5 (89.73) | 49.95 (0.13) | 17.53 (1.34) | 18.78 (0.93) | 8.43 (1.02) | 32.73 (1.66) | 185.75 (15.13) | 0.314958 |
| Decatur | 0.75 (0.5) | 2,797.5 (54.92) | 49.45 (0.37) | 14.05 (0.58) | 17.08 (0.76) | 6.8 (0.47) | 23.95 (0.58) | 114.5 (19.69) | 0.579961 |
| Dickinson | 3.5 (1.73) | 18,494.5 (133.19) | 49.7 (0.14) | 16.8 (0.92) | 18.03 (1.56) | 7.83 (0.41) | 35.03 (1.59) | 93 (9.38) | 0.303606 |
| Doniphan | 1 (1.41) | 7,545.25 (99.7) | 49.4 (0.29) | 16.3 (0.36) | 19.1 (0.68) | 7.48 (0.57) | 26.58 (2.65) | 52.5 (0.58) | 0.26795 |
| Douglas | 9 (2.31) | 120,414.5 (1,566.75) | 50.18 (0.05) | 19.9 (0.91) | 13.78 (1.06) | 7.03 (0.21) | 28.43 (2.12) | 70 (4.55) | 0.231136 |
| Edwards | 0.75 (0.96) | 2,826 (39.83) | 48.75 (0.34) | 14.9 (0.48) | 17.83 (1.03) | 8.53 (0.76) | 25.93 (1.71) | 35.5 (0.58) | 0.515732 |
| Elk | 0.25 (0.5) | 2,479.25 (40.74) | 49.45 (0.33) | 13.28 (0.66) | 19.65 (0.54) | 8.98 (0.56) | 24.53 (4.06) | 59.75 (22.81) | 0.418535 |
| Ellis | 2.5 (1.29) | 28,751.5 (183.71) | 49.55 (0.06) | 19.13 (0.37) | 15.63 (1.23) | 7.18 (0.35) | 29.8 (1.88) | 189.5 (17.54) | 0.486006 |
| Ellsworth | 2 (1.41) | 6,241 (128.94) | 43.05 (0.53) | 17.5 (0.96) | 17.9 (0.89) | 7.78 (0.74) | 28.83 (2.16) | 77.25 (9.54) | 0.408491 |
| Finney | 4 (2) | 37,349 (987.6) | 48.48 (0.1) | 16.6 (1.14) | 18.15 (0.87) | 8.4 (1.21) | 39.13 (2.08) | 103.25 (11.87) | 0.662924 |
| Ford | 2.75 (0.96) | 33,976 (283.79) | 48 (0.12) | 16.95 (0.9) | 18.1 (1.13) | 9.33 (0.74) | 35.48 (3.56) | 69 (5.6) | 0.556725 |
| Franklin | 6.25 (3.3) | 25,802 (253.34) | 49.9 (0.23) | 16.93 (0.43) | 18.58 (0.69) | 7.78 (0.46) | 34.45 (3.58) | 39 (4.62) | 0.292242 |
| Geary | 2.75 (0.5) | 34,336 (2,494.32) | 47.28 (0.22) | 19.08 (0.67) | 18.6 (0.89) | 9.93 (1.18) | 35.78 (3.11) | 267.25 (11.73) | 0.257686 |
| Gove | 0.5 (0.58) | 2,678.25 (65.53) | 49.65 (0.29) | 15.13 (0.69) | 16.88 (0.59) | 7.98 (0.99) | 23.15 (0.73) | 47.75 (19.5) | 0.73972 |
| Graham | 0.25 (0.5) | 2,441.5 (42.84) | 50.45 (0.5) | 13.75 (0.74) | 17.53 (1.04) | 6.8 (0.5) | 26.48 (2.45) | 91.75 (40.19) | 0.595951 |
| Grant | 2 (1.15) | 7,276.25 (85.99) | 49.35 (0.48) | 16.6 (1.49) | 16.7 (1.16) | 6.6 (0.67) | 26.58 (2.45) | 55.75 (23.68) | 0.614368 |
| Gray | 0.5 (0.58) | 5,841.25 (223.33) | 49.13 (0.51) | 16.95 (0.58) | 17.13 (1.13) | 6.25 (0.91) | 29.8 (2.02) | 33.5 (0.58) | 0.614931 |
| Greeley | 0.25 (0.5) | 1,254.75 (28.22) | 50.38 (1.47) | 15.4 (0.71) | 16.43 (1.03) | 6.23 (0.31) | 20.5 (2.05) | 326.25 (5.8) | 0.651618 |
| Greenwood | 2.25 (0.96) | 5,972.25 (37.55) | 49.55 (0.65) | 14.5 (0.63) | 18.53 (0.54) | 8.05 (0.52) | 28.3 (1.78) | 75 (8.68) | 0.393794 |
| Hamilton | 0.75 (0.96) | 2,526 (56.18) | 47.83 (0.26) | 16.4 (0.75) | 18.85 (1.52) | 7.53 (0.74) | 20.25 (2.32) | 49.25 (21.84) | 0.607017 |
| Harper | 1.25 (1.26) | 5,440.75 (66.11) | 49.33 (0.73) | 14.98 (0.84) | 19.7 (0.53) | 7.25 (0.68) | 27.28 (2.71) | 265 (6.22) | 0.395678 |
| Harvey | 4.75 (2.06) | 34,101.75 (268.66) | 50.43 (0.26) | 15.73 (0.67) | 15.83 (1.01) | 6.98 (0.4) | 30.78 (3.75) | 117.75 (9.91) | 0.272506 |
| Haskell | 1 (1.41) | 3,842.75 (163.53) | 49.58 (0.21) | 15.65 (0.71) | 19.23 (1.57) | 6.53 (0.49) | 24.28 (2.12) | 106.75 (13.52) | 0.620082 |
| Hodgeman | 0.5 (0.58) | 1,769.5 (46.26) | 49.1 (0.74) | 15.05 (0.74) | 15.33 (0.71) | 7.13 (0.56) | 24.1 (1.3) | 208 (56.06) | 0.537858 |
| Jackson | 1 (0.82) | 13,235.75 (45.35) | 49.65 (0.31) | 16.2 (0.27) | 18.5 (0.76) | 8.55 (0.93) | 33.4 (1.35) | 66.25 (9.6) | 0.298905 |
| Jefferson | 2 (0.82) | 18,664.75 (340.52) | 49.05 (0.13) | 16.85 (0.66) | 17.4 (0.91) | 8.63 (0.43) | 31.65 (0.78) | 39.5 (2.89) | 0.259697 |
| Jewell | 0.75 (0.96) | 2,900 (46.6) | 48.43 (0.5) | 13.55 (0.53) | 18.43 (1.15) | 7.25 (0.48) | 24.9 (1.97) | 70 (0.82) | 0.464825 |
| Johnson | 70.75 (12.04) | 606,662.75 (7,748.84) | 50.73 (0.21) | 18.15 (0.7) | 11.35 (1.07) | 6.5 (0.18) | 27.7 (1.9) | 118 (10.42) | 0.193535 |
| Kearny | 0.75 (0.96) | 3,908.5 (54.35) | 49.88 (0.48) | 15.68 (0.85) | 19.9 (1.16) | 7.28 (0.5) | 26.03 (4.22) | 233 (26.75) | 0.624916 |
| Kingman | 2.5 (2.89) | 7,270 (114.38) | 49.58 (0.5) | 16.23 (1.01) | 16.35 (0.53) | 7.73 (0.49) | 29.4 (2.51) | 163.5 (25.09) | 0.384093 |
| Kiowa | 0.25 (0.5) | 2,456 (35.87) | 50.55 (0.34) | 15.6 (0.62) | 16.55 (0.94) | 8.05 (0.93) | 25.48 (3.34) | 161.25 (1.71) | 0.506696 |
| Labette | 5.25 (1.26) | 19,898 (221.52) | 49.93 (0.29) | 15.53 (0.56) | 20.45 (0.79) | 9.88 (0.38) | 33.23 (5.38) | 92 (13.88) | 0.344488 |
| Lane | 0 (0) | 1,553.75 (16.13) | 49.9 (0.42) | 15.2 (0.5) | 16.9 (0.75) | 6.95 (0.37) | 25.18 (4.66) | 227 (68.01) | 0.711955 |
| Leavenworth | 9.75 (2.87) | 81,902.75 (379.16) | 46.75 (0.06) | 18.45 (0.4) | 17.4 (1.21) | 10.73 (0.6) | 35.85 (1.71) | 88.75 (9.11) | 0.229788 |
| Lincoln | 0 (0) | 2,953.25 (37.73) | 49.7 (0.56) | 14.8 (0.61) | 16.93 (0.55) | 6.83 (0.9) | 25.7 (1.39) | 108.25 (17.19) | 0.435362 |
| Linn | 1.75 (1.5) | 9,702.5 (79.44) | 49.13 (0.36) | 15 (0.95) | 20.05 (1.29) | 8.65 (0.87) | 31.03 (0.79) | 56.75 (6.08) | 0.303871 |
| Logan | 0 (0) | 2,765.5 (45.6) | 49.43 (0.91) | 16.03 (0.64) | 17.7 (0.58) | 7.45 (0.48) | 23.88 (2.37) | 188 (21.37) | 0.736873 |
| Lyon | 4 (1.41) | 32,660.5 (679.93) | 51.15 (0.29) | 17.13 (0.5) | 16.55 (0.83) | 7.7 (0.49) | 36.55 (1.63) | 81.25 (14.5) | 0.294601 |
| Marion | 3 (2.16) | 11,834.5 (83.12) | 50 (0.35) | 15.38 (0.35) | 16.48 (0.87) | 7.6 (0.34) | 28.95 (3.56) | 67.5 (12.97) | 0.306048 |
| Marshall | 1.5 (1.73) | 9,866.75 (179.87) | 49.83 (0.5) | 15.93 (1.07) | 18 (0.47) | 7.58 (1.14) | 33.5 (1.88) | 59.5 (5) | 0.335113 |
| McPherson | 5.5 (1.91) | 29,355.25 (947.19) | 50.65 (0.3) | 16.15 (0.24) | 16.13 (0.73) | 8.35 (0.69) | 31.05 (3.35) | 63.75 (11.73) | 0.330101 |
| Meade | 1.25 (1.5) | 4,047.75 (42.51) | 48.85 (0.42) | 15.93 (0.79) | 17.73 (0.81) | 7.5 (0.95) | 24.55 (4.25) | 188.25 (35.36) | 0.602431 |
| Miami | 4 (1.41) | 34,208.5 (369.82) | 50.03 (0.28) | 17.48 (0.46) | 16.1 (0.76) | 7.1 (0.37) | 35.15 (3.97) | 62.5 (9.57) | 0.262556 |
| Mitchell | 0.5 (0.58) | 5,904.25 (170.93) | 49.18 (0.43) | 15.68 (0.57) | 16.7 (0.63) | 7.38 (0.63) | 30.33 (3.95) | 41.75 (10.11) | 0.469074 |
| Montgomery | 6.75 (1.71) | 31,625 (411.27) | 50.25 (0.19) | 14.68 (1.28) | 20.13 (1.33) | 9.83 (0.32) | 38.08 (1.58) | 134.5 (10.88) | 0.346967 |
| Morris | 0.5 (0.58) | 5,483.75 (123.88) | 49.8 (0.18) | 15.63 (0.88) | 17.3 (0.89) | 7.5 (0.86) | 27.18 (2.03) | 49.25 (26.73) | 0.325753 |
| Morton | 0.5 (1) | 2,664.25 (52.89) | 49.95 (0.33) | 15.25 (0.7) | 18.05 (0.89) | 7.2 (0.34) | 22.58 (1.41) | 113.25 (27.42) | 0.611152 |
| Nemaha | 2.25 (1.5) | 10,211.75 (59.96) | 49.03 (0.43) | 16.38 (1.03) | 16.35 (0.92) | 5.58 (0.29) | 27.63 (2.56) | 83.75 (25.24) | 0.337378 |
| Neosho | 2.5 (1.29) | 15,932 (107.83) | 50.1 (0.37) | 15.78 (1.03) | 18.85 (0.66) | 8.9 (1.28) | 32.8 (1.45) | 140.75 (21.53) | 0.360799 |
| Ness | 0.25 (0.5) | 2,720.75 (63.77) | 50.63 (0.5) | 14.63 (0.92) | 16.75 (0.81) | 8.18 (0.57) | 27.3 (0.61) | 62.5 (18.38) | 0.615745 |
| Norton | 0.25 (0.5) | 5,400.5 (42.76) | 43.43 (0.9) | 17.48 (0.69) | 18.4 (1.09) | 7.98 (0.25) | 24.58 (1.89) | 51 (8.68) | 0.535586 |
| Osage | 3.5 (1) | 15,845.25 (113.68) | 49.68 (0.26) | 16.23 (0.86) | 18.93 (0.99) | 9.33 (1.25) | 39.28 (4.11) | 45.75 (3.5) | 0.279687 |
| Osborne | 1.25 (1.26) | 3,464.5 (32.31) | 49.2 (0.22) | 14.95 (0.17) | 17.2 (0.42) | 7.6 (0.7) | 29.55 (1.25) | 86 (2.16) | 0.536959 |
| Ottawa | 2.25 (0.5) | 5,755.5 (56.52) | 47.88 (0.26) | 16.1 (0.47) | 17.43 (0.4) | 7.63 (0.46) | 28.8 (2.97) | 26 (10.39) | 0.393014 |
| Pawnee | 0.5 (0.58) | 6,368.75 (158.03) | 44.23 (0.46) | 16.73 (0.39) | 18.73 (1.2) | 8.08 (0.35) | 30.93 (1.02) | 197 (51.59) | 0.479734 |
| Phillips | 0.5 (0.58) | 5,071.75 (219.96) | 50 (0.41) | 15.3 (0.47) | 17.58 (0.63) | 7.05 (0.51) | 29.1 (2.51) | 114 (17.17) | 0.508159 |
| Pottawatomie | 4 (2.45) | 24,974.25 (808.79) | 50.1 (0.23) | 18.05 (0.7) | 15.83 (1.07) | 8.05 (0.17) | 33.2 (1.16) | 40 (9.45) | 0.28301 |
| Pratt | 2 (1.41) | 9,202.75 (92.4) | 50 (0.5) | 16.35 (0.79) | 16.73 (1.07) | 7.35 (0.95) | 28.63 (2.8) | 172.5 (29.44) | 0.432356 |
| Rawlins | 0.25 (0.5) | 2,541.75 (32.29) | 49.23 (0.31) | 14.35 (0.24) | 16.73 (0.66) | 6.48 (0.17) | 21.8 (2.95) | 109.75 (19.84) | 0.631495 |
| Reno | 9.25 (3.5) | 61,936.5 (332.77) | 49.58 (0.21) | 15.08 (0.48) | 18.6 (0.77) | 9.9 (0.92) | 38.4 (3.54) | 117.25 (14.45) | 0.339447 |
| Republic | 1 (0.82) | 4,664.75 (27.17) | 50.28 (0.22) | 14.5 (0.54) | 16.03 (0.45) | 6.9 (0.95) | 29.88 (0.42) | 113.5 (12.34) | 0.396426 |
| Rice | 2.5 (1.91) | 9,448.25 (74.64) | 49.48 (0.56) | 16.55 (0.72) | 16.95 (1.05) | 8.18 (0.67) | 32.1 (1.28) | 55 (6) | 0.368607 |
| Riley | 5.75 (2.06) | 73,206.25 (1,475.76) | 47 (0.36) | 21.45 (1.84) | 14.6 (1.14) | 7 (0.29) | 25.9 (1.79) | 120.5 (6.56) | 0.268179 |
| Rooks | 0.25 (0.5) | 4,915 (58.88) | 50.68 (1.03) | 15.7 (0.43) | 17.6 (0.5) | 6.75 (0.31) | 24.6 (2.83) | 60.5 (15.55) | 0.5233 |
| Rush | 0.5 (0.58) | 2,992.75 (62.14) | 48.63 (0.3) | 14.95 (0.69) | 16.98 (0.78) | 6.5 (0.73) | 27.35 (3.03) | 189.25 (31.08) | 0.484902 |
| Russell | 1 (1.15) | 6,781.25 (114.74) | 50.38 (0.5) | 15.2 (0.61) | 17.6 (0.58) | 6.98 (0.54) | 29.75 (0.89) | 145.75 (13.07) | 0.478437 |
| Saline | 5 (1.63) | 54,172 (239.69) | 50.25 (0.17) | 15.75 (0.61) | 17.6 (0.79) | 8.3 (0.54) | 34.63 (2.17) | 118.75 (17.75) | 0.337186 |
| Scott | 0.25 (0.5) | 4,995 (153.21) | 49.28 (0.38) | 15.73 (0.43) | 17.55 (1.1) | 7.13 (0.62) | 24.33 (2.77) | 113 (10.42) | 0.655398 |
| Sedgwick | 59.5 (5.2) | 519,708.25 (5,806.92) | 50.43 (0.15) | 15.7 (0.73) | 17.93 (0.78) | 10.2 (0.14) | 36.5 (2.06) | 146.75 (11.24) | 0.236211 |
| Seward | 3 (0.82) | 21,737.75 (214.35) | 48.85 (0.31) | 16.03 (1.11) | 18.55 (1.21) | 7.75 (0.78) | 32.2 (1.78) | 86 (19.13) | 0.550807 |
| Shawnee | 29.25 (3.3) | 177,825 (881.76) | 51.45 (0.17) | 15 (1.04) | 17.25 (1.14) | 10.28 (0.59) | 37.98 (0.67) | 207 (19.92) | 0.219594 |
| Sheridan | 0.25 (0.5) | 2,484.25 (43.14) | 49.43 (0.3) | 15.85 (1) | 16.23 (1.11) | 6.53 (0.3) | 24.23 (3.44) | 197.75 (32.66) | 0.638937 |
| Sherman | 0.25 (0.5) | 5,918.75 (15.24) | 49.25 (0.75) | 16.6 (0.98) | 16.78 (1.47) | 6.8 (0.89) | 30.33 (1.19) | 157.5 (10.34) | 0.59156 |
| Smith | 0.5 (1) | 3,574.5 (14.43) | 49.85 (0.31) | 14.13 (0.7) | 16.75 (1) | 6.78 (0.96) | 26.55 (0.6) | 77 (27.51) | 0.491588 |
| Stafford | 1 (2) | 4,095.5 (75.16) | 49.2 (0.08) | 15.1 (0.64) | 17.4 (0.83) | 7.33 (0.46) | 26.1 (2.18) | 54.25 (11.84) | 0.438313 |
| Stanton | 0 (0) | 2,040.5 (33.87) | 50.38 (0.36) | 15.38 (0.46) | 17.53 (1.12) | 6.4 (0.29) | 21.58 (2.57) | 75.25 (29.17) | 0.586672 |
| Stevens | 0.75 (0.5) | 5,393 (139.36) | 50.7 (0.18) | 15.68 (0.5) | 19.05 (0.79) | 7.73 (0.78) | 28.75 (2.11) | 54.5 (1.29) | 0.531164 |
| Sumner | 4 (0.82) | 22,626.25 (355.79) | 49.8 (0.12) | 15.75 (0.77) | 18.53 (0.88) | 8.4 (0.48) | 35 (2.63) | 48 (3.74) | 0.311916 |
| Thomas | 1.5 (1.73) | 7,842.5 (117.75) | 50.75 (0.44) | 17.48 (0.42) | 16.48 (0.75) | 7.63 (0.72) | 28.4 (2.62) | 190.75 (7.23) | 0.632605 |
| Trego | 0.25 (0.5) | 2,798.75 (6.02) | 49 (0.18) | 15.28 (0.71) | 16.33 (0.61) | 7.9 (0.92) | 24.28 (2.11) | 168.75 (16.05) | 0.6095 |
| Wabaunsee | 2 (2.16) | 6,925.75 (33.93) | 48.83 (0.1) | 16.88 (0.79) | 16 (0.68) | 7.58 (0.7) | 29.43 (1.93) | --- | 0.289409 |
| Wallace | 0 (0) | 1,508 (12.94) | 49.75 (0.31) | 15.35 (0.64) | 15.38 (0.77) | 6.45 (0.26) | 19.93 (1.46) | 66 (0.82) | 0.645672 |
| Washington | 0 (0) | 5,475.75 (58.03) | 48.8 (0.29) | 15.63 (0.67) | 17.18 (0.39) | 6.55 (0.34) | 26.78 (4.79) | 55 (0) | 0.358337 |
| Wichita | 0 (0) | 2,114.75 (28.73) | 47.63 (1.23) | 15.68 (0.39) | 15.63 (1.24) | 7.85 (0.31) | 28.3 (2.84) | 154.75 (46.42) | 0.652407 |
| Wilson | 0.75 (0.96) | 8,575.5 (66.84) | 50.2 (0.35) | 15.15 (0.58) | 20.28 (0.97) | 8.83 (0.59) | 30.08 (1.96) | 134.5 (13.7) | 0.394019 |
| Woodson | 1 (1.41) | 3,126.75 (44.1) | 49.35 (0.47) | 14.43 (0.72) | 19.93 (1.96) | 7.4 (0.5) | 23.7 (1.85) | 0 (0) | 0.380066 |
| Wyandotte | 13 (1.83) | 166,737.25 (1,710.28) | 50 (0.36) | 16.23 (0.92) | 22.2 (1.4) | 12.6 (0.68) | 41.78 (1.13) | 125.75 (9.71) | 0.178355 |
