# Supplementary figures and images for: Spatial and temporal modeling of breast cancer mortality in Kansas: An R-INLA approach
Source: PLoS One. 2026 Apr 29;21(4):e0347607. doi: 10.1371/journal.pone.0347607 (PMC13127976; doi:10.1371/journal.pone.0347607)

**S2:** Variation in Average Demographics Across Kansas Counties (2018–2021)

**
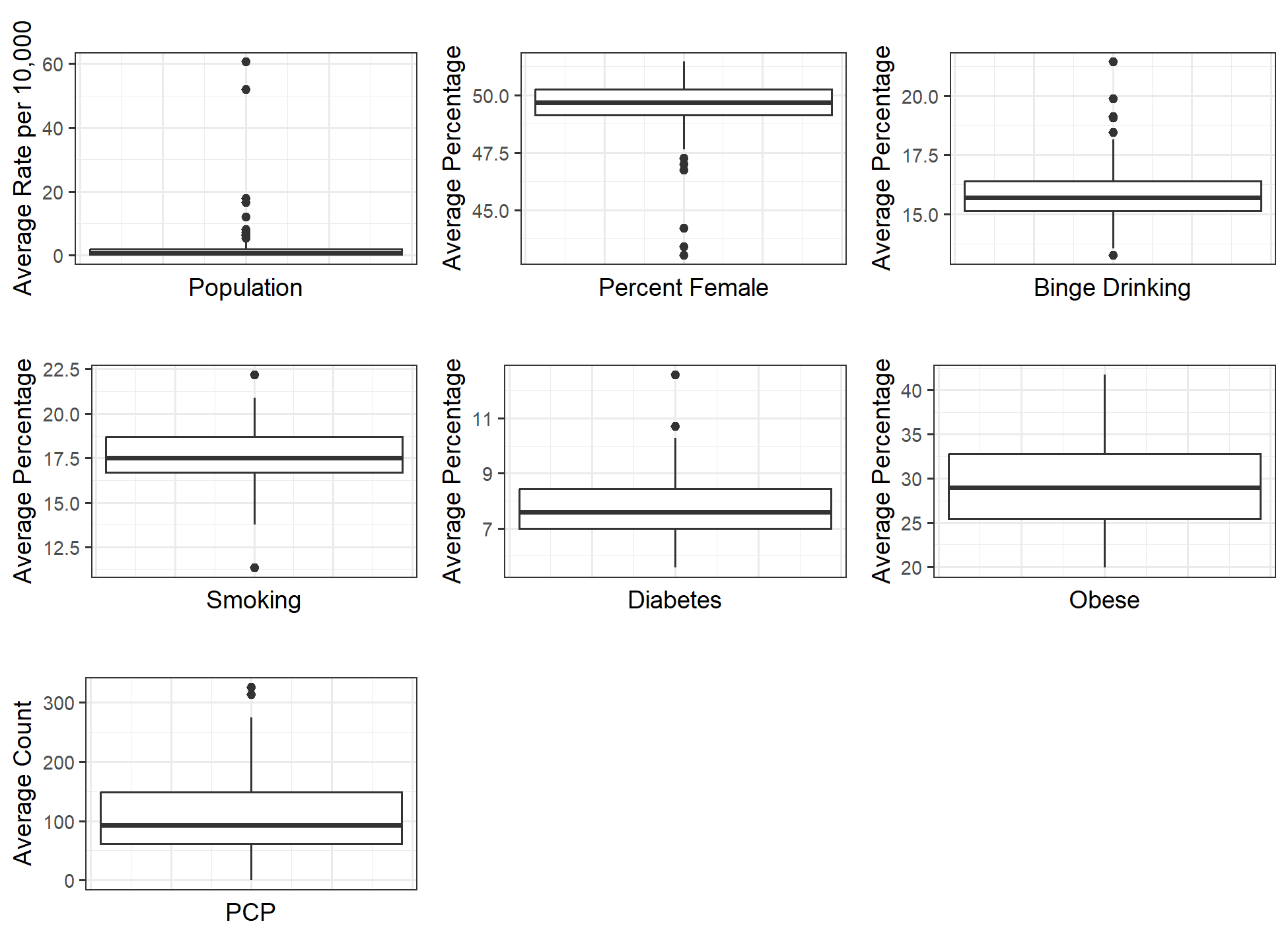
**

Supplement: S2 Fig — (DOCX) [file pone.0347607.s002.docx]

**S4:** Correlation Matrix


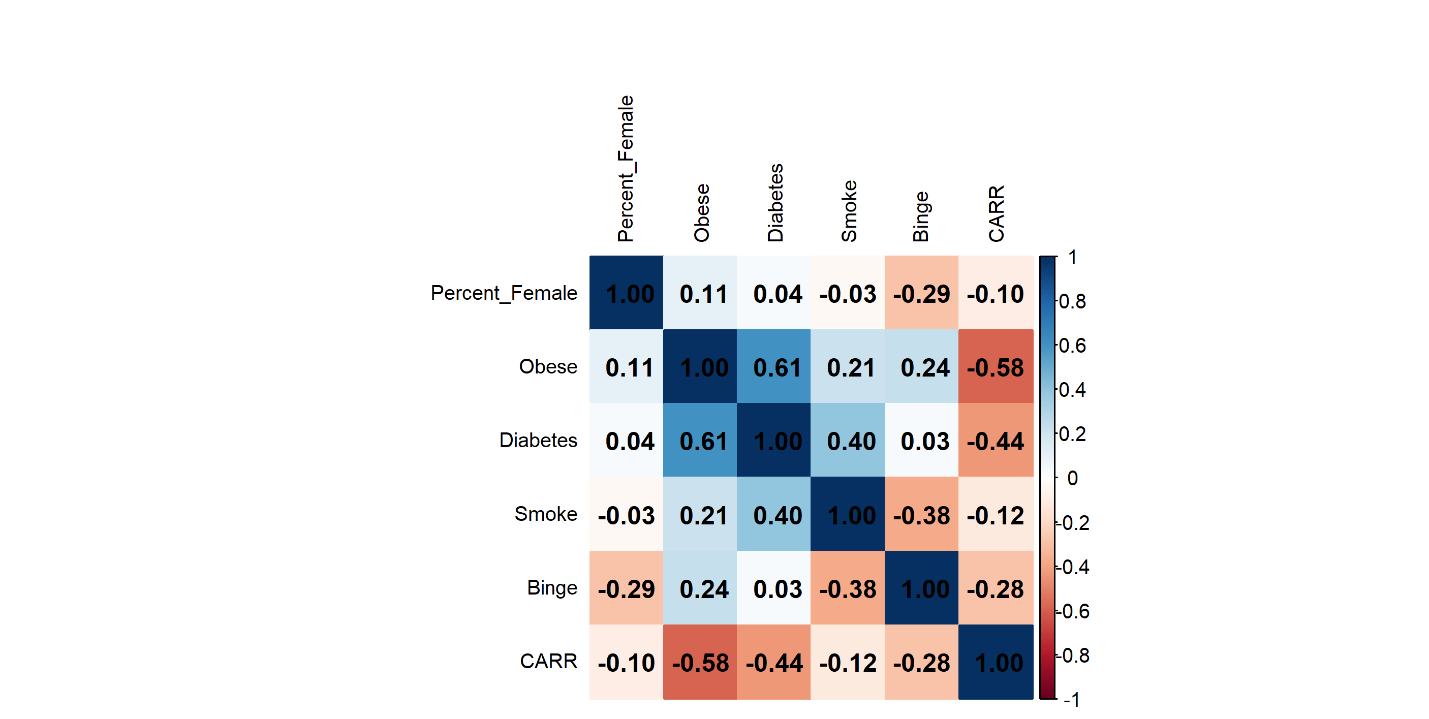

Supplement: S4 Fig — (DOCX) [file pone.0347607.s004.docx]
