# Supplementary material for: Spatial and temporal modeling of breast cancer mortality in Kansas: An R-INLA approach
Source: PLoS One. 2026 Apr 29;21(4):e0347607. doi: 10.1371/journal.pone.0347607 (PMC13127976; doi:10.1371/journal.pone.0347607)
Supplement: S3 Table — (DOCX) [file pone.0347607.s003.docx]

**S3:** Cluster Membership Table

| **County Name** | **Cluster Membership** | **County Name** | **Cluster Membership** | **County Name** | **Cluster Membership** | **County Name** | **Cluster Membership** |
| --- | --- | --- | --- | --- | --- | --- | --- |
| Barton County | Central | Atchison County | Metro | Cheyenne County | Northwest | Barber County | Southwest |
| Butler County | Central | Brown County | Metro | Clay County | Northwest | Clark County | Southwest |
| Chase County | Central | Doniphan County | Metro | Decatur County | Northwest | Comanche County | Southwest |
| Chautauqua County | Central | Jackson County | Metro | Ellis County | Northwest | Edwards County | Southwest |
| Cloud County | Central | Jefferson County | Metro | Gove County | Northwest | Finney County | Southwest |
| Cowley County | Central | Leavenworth County | Metro | Graham County | Northwest | Ford County | Southwest |
| Dickinson County | Central | Wyandotte County | Metro | Greeley County | Northwest | Grant County | Southwest |
| Elk County | Central | Douglas County | Northeast | Hodgeman County | Northwest | Gray County | Southwest |
| Ellsworth County | Central | Johnson County | Northeast | Jewell County | Northwest | Hamilton County | Southwest |
| Geary County | Central | Miami County | Northeast | Lane County | Northwest | Harper County | Southwest |
| Greenwood County | Central | Allen County | Southeast | Lincoln County | Northwest | Haskell County | Southwest |
| Harvey County | Central | Anderson County | Southeast | Logan County | Northwest | Kearny County | Southwest |
| Marion County | Central | Bourbon County | Southeast | Mitchell County | Northwest | Kingman County | Southwest |
| Marshall County | Central | Cherokee County | Southeast | Ness County | Northwest | Kiowa County | Southwest |
| McPherson County | Central | Coffey County | Southeast | Norton County | Northwest | Meade County | Southwest |
| Morris County | Central | Crawford County | Southeast | Osborne County | Northwest | Morton County | Southwest |
| Nemaha County | Central | Franklin County | Southeast | Ottawa County | Northwest | Pratt County | Southwest |
| Pottawatomie County | Central | Labette County | Southeast | Pawnee County | Northwest | Seward County | Southwest |
| Reno County | Central | Linn County | Southeast | Phillips County | Northwest | Stafford County | Southwest |
| Republic County | Central | Lyon County | Southeast | Rawlins County | Northwest | Stanton County | Southwest |
| Rice County | Central | Montgomery County | Southeast | Rooks County | Northwest | Stevens County | Southwest |
| Riley County | Central | Neosho County | Southeast | Rush County | Northwest |  |  |
| Saline County | Central | Osage County | Southeast | Russell County | Northwest |  |  |
| Sedgwick County | Central | Shawnee County | Southeast | Scott County | Northwest |  |  |
| Sumner County | Central | Wilson County | Southeast | Sheridan County | Northwest |  |  |
| Wabaunsee County | Central |  |  | Sherman County | Northwest |  |  |
| Washington County | Central |  |  | Smith County | Northwest |  |  |
| Woodson County | Central |  |  | Thomas County | Northwest |  |  |
|  |  |  |  | Trego County | Northwest |  |  |
|  |  |  |  | Wallace County | Northwest |  |  |
|  |  |  |  | Wichita County | Northwest |  |  |
