# Supplementary material for: Spatial and temporal modeling of breast cancer mortality in Kansas: An R-INLA approach
Source: PLoS One. 2026 Apr 29;21(4):e0347607. doi: 10.1371/journal.pone.0347607 (PMC13127976; doi:10.1371/journal.pone.0347607)
Supplement: S5 File — (DOCX) [file pone.0347607.s005.docx]

**S5:** Sensitivity analysis for hotspot thresholds

County- and cluster-level exceedance probabilities under RR>2.0, probability >0.80, and RR>1.5, probability >0.70).

***County-level Sensitivity analysis for Exceedance Probabilities***

**Scenario 1: RR>1.5, p>0.70**


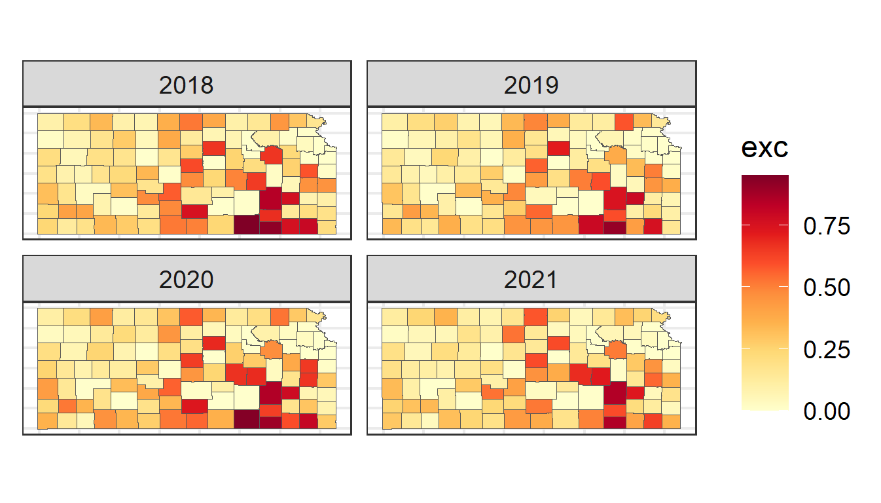


| **Year** | **County** | **Exceedance Probability** |
| --- | --- | --- |
| 2018 | Kingman County | 0.7553974 |
| 2018 | Montgomery County | 0.7625143 |
| 2018 | Woodson County | 0.765509 |
| 2018 | Labette County | 0.8005101 |
| 2018 | Greenwood County | 0.8468886 |
| 2018 | Chautauqua County | 0.9219487 |
| 2018 | Cowley County | 0.9520435 |
| 2019 | Ottawa County | 0.7136085 |
| 2019 | Greenwood County | 0.7486937 |
| 2019 | Labette County | 0.7606913 |
| 2019 | Woodson County | 0.7928 |
| 2019 | Cowley County | 0.7947091 |
| 2019 | Chautauqua County | 0.9083248 |
| 2020 | Kingman County | 0.7372116 |
| 2020 | Woodson County | 0.7862681 |
| 2020 | Labette County | 0.8082182 |
| 2020 | Greenwood County | 0.8556285 |
| 2020 | Chautauqua County | 0.8954306 |
| 2020 | Cowley County | 0.943752 |
| 2021 | Chase County | 0.7246304 |
| 2021 | Woodson County | 0.7279853 |
| 2021 | Greenwood County | 0.8532578 |
| 2021 | Chautauqua County | 0.8568302 |

**Scenario 2: RR>2.0, p>0.80**


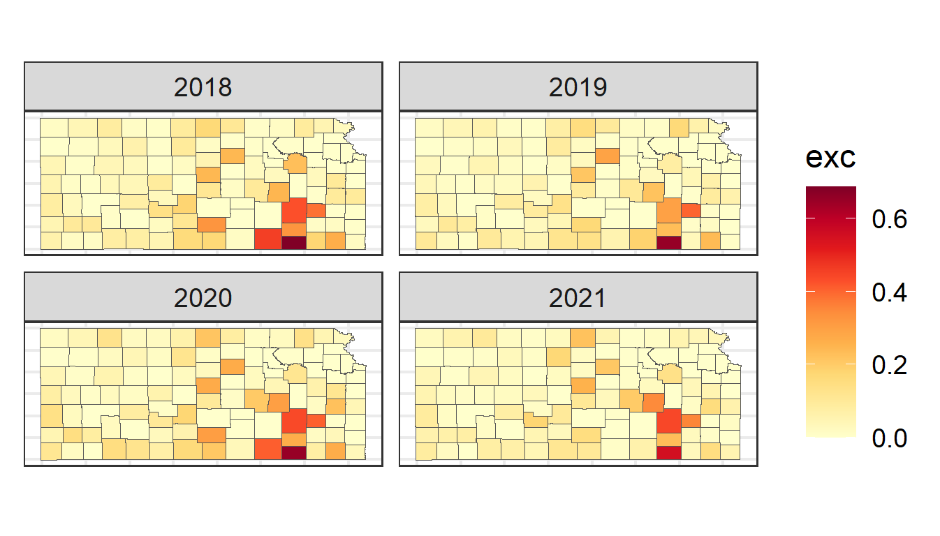


**NOTE:** There were no counties in any year that had exceedance probability greater than 0.80.

***Cluster-level Sensitivity analysis for Exceedance Probabilities***

**Scenario 1: *RR>1.50, p>0.70***


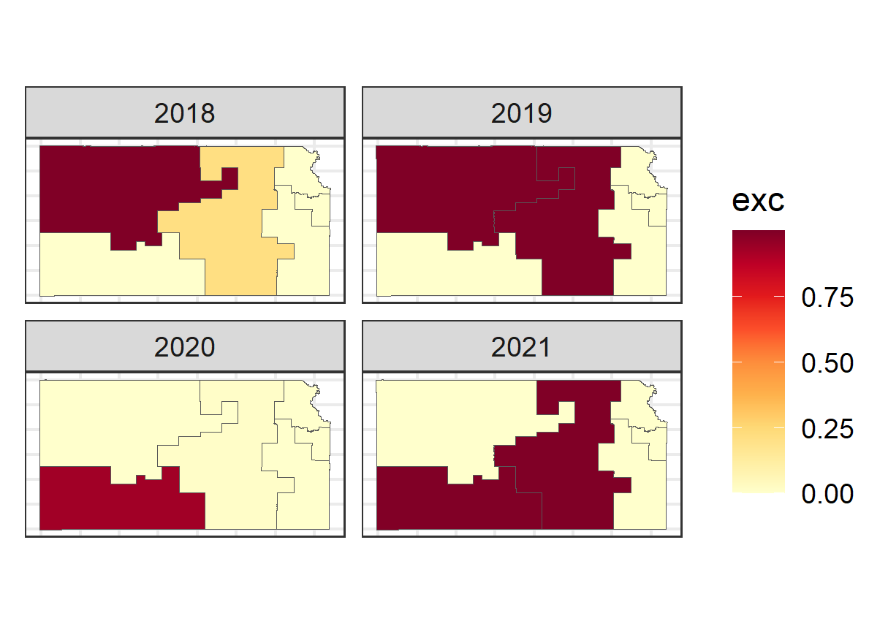


| **Year** | **Cluster** | **Exceedance Probability** |
| --- | --- | --- |
| 2018 | Northwest | 1.000 |
| 2019 | Northwest | 1.000 |
|  | Central | 1.000 |
| 2020 | Southwest | 0.931 |
| 2021 | Central | 1.000 |
|  | Southwest | 0.997 |

**Scenario 2: *RR*>2.0, p>0.80**


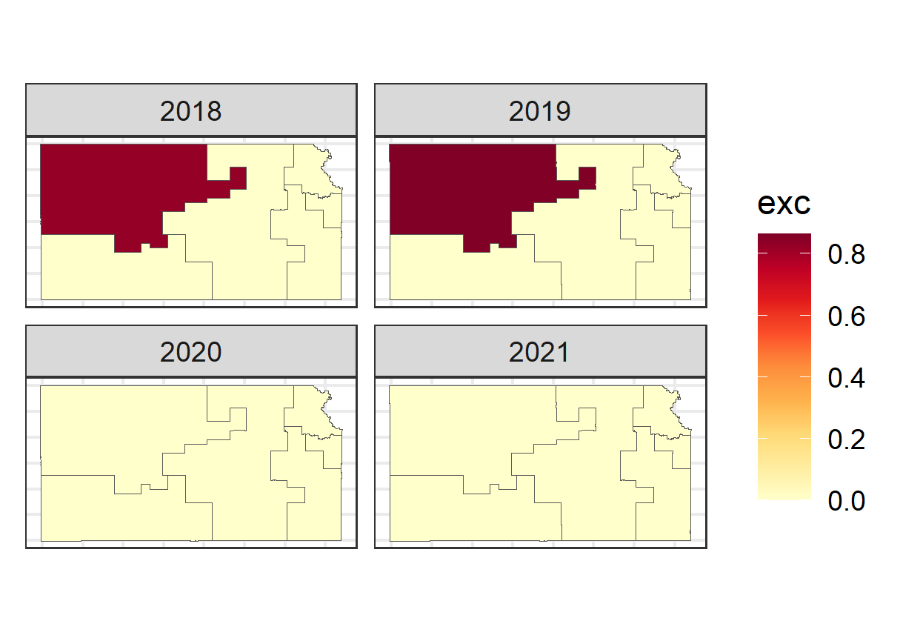


| **Year** | **Cluster** | **Exceedance Probability** |
| --- | --- | --- |
| 2018 | Northwest | 0.825 |
| 2019 | Northwest | 0.862 |
