# Supplementary material for: Spatial and temporal modeling of breast cancer mortality in Kansas: An R-INLA approach
Source: PLoS One. 2026 Apr 29;21(4):e0347607. doi: 10.1371/journal.pone.0347607 (PMC13127976; doi:10.1371/journal.pone.0347607)
Supplement: Attachment — Submitted filename: Final Review Report.docx [file pone.0347607.s006.docx]

# Reviewer Report – PLOS ONE

Thank you for the opportunity to review this manuscript. The topic—spatial and temporal variation in breast cancer mortality across Kansas—is relevant to public health and appropriate for the scope of PLOS ONE. The use of Bayesian spatial models and R-INLA is suitable for small-area mortality data, and the authors make a clear effort to compare multiple modeling approaches.

However, in its current form, the manuscript requires major revision. While the general modeling framework is sound, several issues related to interpretation, methodological clarity, and internal consistency limit the reliability of the conclusions.

Major comments

1. Outcome model, offset, and interpretation of relative risk

Although the manuscript repeatedly reports county- and cluster-level “relative risks,” it is not clearly specified what these risks are relative to. The model description alternates between population offsets and expected counts, but the practical implementation is not explicitly stated.

The authors should clearly specify whether the model uses (i) observed counts with a log(population) offset, (ii) observed versus expected counts with internally standardized expected values, or another formulation. If expected counts are used, the manuscript must describe how they were calculated, including whether age-standardization was applied and what reference rates were used.

2. Temporal modeling with only four years of data

The study period includes only four years (2018–2021), which severely limits the identifiability of temporal trends. While the county-level analysis finds no statistically significant time effect, the cluster analysis suggests temporal variation, leading to potentially conflicting interpretations.

The authors should justify the choice of temporal structure given the short time series, clarify the priors used for temporal effects, and consider presenting a simpler alternative such as year-specific fixed effects. At a minimum, the limitations of interpreting temporal “trends” over four years should be stated more explicitly.

3. Spatial clustering procedure and reproducibility

The manuscript states that clusters were constructed using 2018 data and assumed constant over time, which is a consequential modeling choice. While the SKATER algorithm is described conceptually, key implementation details needed for replication are missing.

The authors should clearly specify the variables used for clustering, how they were scaled, the distance metric employed, the contiguity definition, the number of clusters selected, and the criteria used to choose that number. Cluster membership should be reported, either in a table or supplement, along with a clear cluster map.

Additionally, the manuscript should explain why certain covariates become statistically significant in the cluster analysis but not in the county-level analysis, and whether this reflects aggregation effects, variance reduction, or ecological bias.

4. Interpretation of binge drinking coefficient

In the county-level model, the coefficient for percent female binge drinking is negative and statistically significant, implying that higher binge drinking prevalence is associated with lower breast cancer mortality risk. However, the discussion interprets this variable as a positive risk factor.

This discrepancy must be resolved. If the coefficient is correctly estimated, the discussion should acknowledge and explain this counterintuitive association, possibly in terms of confounding, urban–rural structure, or correlated covariates. Alternatively, if the variable was transformed or coded in a nonstandard way, this must be clearly stated. Presenting a correlation matrix or multicollinearity diagnostics would strengthen this section.

5. Model comparison language

While the manuscript correctly notes that many models have similar DIC and WAIC values, it still repeatedly labels the BYM2 Poisson model as the “best” model. This language should be softened and made consistent.

The authors should state explicitly that the BYM2 Poisson model was selected primarily for interpretability and parsimony, while acknowledging that several competing models fit the data comparably. Given the large number of zero counts, an additional posterior predictive check or brief discussion of overdispersion would further strengthen the analysis.

6. Hotspot definition and sensitivity

Hotspots are identified using exceedance probabilities with thresholds of RR > 1.5 and probability > 0.75. While reasonable, these thresholds are not justified.

The authors should provide a brief rationale for these choices and include a simple sensitivity analysis showing how hotspot identification changes under alternative thresholds. This would improve the robustness and credibility of the hotspot findings.

7. Results inconsistencies

There are several technical inconsistencies, including statements implying that relative risk can be less than zero and unexplained extreme intercept values in cluster-level models. County-level and cluster-level results should be presented in a more parallel manner.

8. Discussion alignment

The Discussion relies heavily on background literature and does not sufficiently engage with the study’s own findings. Unexpected results should be addressed directly, ecological limitations integrated more clearly, and policy implications stated more cautiously.

Minor Comments

• Several sentences are overly long and could be simplified.

• Minor grammatical issues are present, including subject–verb agreement errors, inconsistent capitalization, and hyphenation. This need a total proofread.

**Final Recommendation**

**Major Revision**
