# Supplementary material for: Spatial and temporal modeling of breast cancer mortality in Kansas: An R-INLA approach
Source: PLoS One. 2026 Apr 29;21(4):e0347607. doi: 10.1371/journal.pone.0347607 (PMC13127976; doi:10.1371/journal.pone.0347607)
Supplement: Attachment — Submitted filename: PONE-D-25-63477 point-by-point response.docx [file pone.0347607.s009.docx]

March 28, 2026

Majid Bani-Yaghoub

Academic Editor

PLOS One

On behalf of my co-authors, we appreciate the opportunity to revise and resubmit our manuscript, “*Spatial and Temporal Modeling of Breast Cancer Mortality in Kansas: An R-INLA Approach*” (Submission ID PONE-D-25-63477), for consideration as a research article in PLOS One. The reviewer's comments were highly constructive and strengthened our manuscript. We thank all the reviewers and the Editor for their advice and suggestions.

In the pages that follow, we provide point-by-point responses. We indicated our responses in blue text. We again note that this manuscript has been submitted only to *PLOS One,* has not been published nor submitted elsewhere, and that all research adheres to the journal’s ethical guidelines. We look forward to your hopefully positive decision on this manuscript. Thank you again for your consideration.

Sincerely,

Dinesh Pal Mudaranthakam, PhD, MBA,

Assistant Professor, Department of Biostatistics & Data Science

University of Kansas Medical Center

3901 Rainbow Boulevard

Kansas City, KS 66160

Phone: (913)-945-6922

Email: dmudaranthakam@kumc.edu

We appreciate the time and effort from all reviewers to help us improve our manuscript. The comments and suggestions have helped clarify key points and have strengthened the manuscript. All the authors have edited and approve of the changes made.

**Comments from the Reviewers**

Reviewer #1: I appreciate the opportunity to review the revised manuscript.

Authors have made good changes and updates based on comments and recommendations from reviewers.

I have only a few minor suggestions.

We thank the reviewers for their positive assessment and for acknowledging the improvements made in response to prior comments. We appreciate the additional minor suggestions and have carefully reviewed and addressed them in the revised manuscript. We believe these final refinements further improve the clarity, rigor, and presentation of the study.

If possible, I suggest the authors could create a publicly accessible GitHub repository with readme file, codes and data used for statistical analysis and figures, This would enable reproducibility of the results from the paper. This might also help in understanding how the figures were generated.

We thank the reviewer for this excellent suggestion and fully agree that providing analysis code improves transparency and reproducibility. In response, we created a publicly accessible GitHub repository that includes a detailed README, the analysis scripts, and supporting materials used to generate the statistical results and figures in the manuscript.

The repository is available at: <https://github.com/spepperkumc/Spatial-Temporal-Modeling-INLA>.

To protect confidentiality and comply with data-use and disclosure constraints, any non-shareable or restricted components (if applicable) have been clearly documented in the README, along with instructions for reproducing the analysis workflow and figure generation. We believe this addition substantially strengthens the rigor, transparency, and reproducibility of the study.

Also, if there are any future directions that the author plans to take based on the current work, it could be a good addition especially since this analysis is on a short time series. It will be interesting to know what the future research direction is and if there are plans to look into exploring beyond the county levels.

We thank the reviewer for this thoughtful suggestion and agree that outlining future research directions strengthens the manuscript, particularly given the short temporal window of the current analysis. In response, we have added a brief Future Directions discussion highlighting planned extensions of this work. Specifically, we note the potential to (1) incorporate longer time series as additional mortality data become available to better assess long-term temporal trends, and (2) extend the analysis to finer spatial resolutions (e.g., census tract or sub‑county units) and multi‑level spatial frameworks as data availability and privacy constraints allow. We have also emphasized that future multi‑state or multi‑registry analyses could help evaluate the generalizability of the observed spatial patterns beyond Kansas. These additions clarify the ongoing research trajectory while appropriately distinguishing future work from the scope of the present study.

Overall, the authors have made significant changes to the paper, and I believe the paper could be published.

Reviewer #3: The authors have addressed most of the major concerns raised in the previous review. In particular, the clarification of the outcome model and relative risk interpretation, improved temporal modeling justification, resolution of the binge drinking coefficient interpretation, and addition of hotspot sensitivity analysis substantially strengthen the manuscript.

However, a few issues remain that require clarification before acceptance. Specifically, the spatial clustering procedure still lacks key reproducibility details:

1. Which covariates were used for clustering

2. How many clusters were chosen

3. Criterion for selecting number of clusters

4. Actual cluster membership (table or supplement)

We thank the reviewer for this important comment. We have revised the manuscript to provide additional methodological detail to ensure full transparency and reproducibility of the clustering procedure (pg21-22. Lines 521-560). Specifically, we now clarify that:

- Spatial clustering was based solely on 2018 county-level data.
- The variables used for clustering were percent female, obesity prevalence, diabetes prevalence, smoking prevalence, binge drinking prevalence, number of primary care physicians, and the rurality index (CARR).
- All covariates were standardized to z-scores prior to clustering.
- Euclidean distance was used to compute dissimilarity between neighboring counties.
- Spatial contiguity was defined using first-order queen adjacency.
- Clustering was implemented in R using the skater function within the *spdep* package.
- A minimum case constraint of 21 total breast cancer mortality cases (based on 2018 data) was imposed to ensure statistical stability and confidentiality.
- Six clusters were selected based on the elbow criterion applied to within-cluster sums of squares while satisfying the minimum case constraint.
- A complete listing of county-to-cluster assignments is now provided in Supplementary Table S3.
- A clear cluster map is provided in Fig 6.

We have also added clarification in the Results section explaining that differences in statistical significance between the county-level and cluster-level analyses likely reflect aggregation effects and variance reduction. By combining counties into larger spatial units, case counts increase and variability decreases, improving statistical stability and power to detect associations. However, we acknowledge that aggregation may introduce ecological bias, and cluster-level findings should therefore be interpreted within the ecological framework of the study.

Moreover, the manuscript states that expected counts are computed using internally standardized crude rates. However, breast cancer mortality is strongly age-dependent, and failure to use age-standardized rates may introduce bias in the estimated relative risks.

We thank the reviewer for this important point and agree that breast cancer mortality is strongly age-dependent.

We would like to clarify that our inferential framework is a count-based disease mapping model (Poisson/BYM2 spatiotemporal model), where the outcome is mortality counts and the “baseline risk” is incorporated through an offset using expected counts (i.e., Yit∼Poisson(Eitexp⁡(ηit))Y_{it} \sim \text{Poisson}(E_{it}\exp(\eta_{it}))Yit∼Poisson(Eitexp(ηit))). In this setting, age-standardization is implemented through how EitE_{it}Eit is computed (e.g., indirect standardization using age-specific reference rates), rather than by modeling age-standardized rates as the outcome.

It is better to:

5. Justify the use of crude rates instead of age-standardized expected counts, or

We thank the reviewer for this comment. Crude (internally standardized) expected counts were used due to limited availability and instability of age‑specific population data at the county–year level, particularly in areas with small counts. Our primary aim was to identify relative spatial patterns rather than produce age‑adjusted absolute rates. We have clarified this rationale and its implications for interpretation in the Methods and Limitations sections.

6. Discuss this limitation explicitly and its potential impact on spatial risk interpretation

We thank the reviewer for highlighting the importance of explicitly addressing this limitation. We agree that this issue has important implications for the interpretation of spatial risk estimates. In response, we have revised the Discussion and Limitations sections to explicitly state how data aggregation, small-area counts, and spatial smoothing may influence estimated relative risks and hotspot identification. Specifically, we now clarify that spatial risk estimates reflect population-level associations rather than individual-level risk and that Bayesian smoothing and spatial clustering may attenuate extreme values while improving estimate stability. We also emphasize that identified spatial patterns should be interpreted as hypothesis-generating and reflective of structural and geographic variation, rather than precise measures of localized causal risk. These additions provide clearer guidance on how readers should interpret spatial risk estimates given the underlying data and modeling assumptions.

Addressing these remaining points, particularly the clarification of the clustering procedure and the discussion of age-standardization will further strengthen the rigor, transparency, and reproducibility of the study.

We thank the reviewer for their constructive feedback and for the positive assessment of the revisions. All remaining minor suggestions, including clarification of the clustering procedure and the discussion of age standardization, have been fully incorporated and addressed, further strengthening the rigor, transparency, and reproducibility of the study.

Reviewer #4: All the concerns I had before we addressed. Additionally, the formatting changes to the font and style served well to highlight the papers results and major points.

Also, I thought the reduction in the amount of the equations helped the reader better understand the methods used in generate the equations.

We thank the reviewer for their positive assessment of the revisions. We appreciate the feedback regarding the improved formatting and streamlined presentation of the methods, and we are pleased that these changes enhanced clarity and readability.
